# Three-dimension structure of ventricular myocardial fibers after myocardial infarction

**DOI:** 10.1186/1749-8090-5-116

**Published:** 2010-11-23

**Authors:** Changqing Gao, Weihua Ye, Libin Li

**Affiliations:** 1 Department of Cardiovascular Surgery, PLA General Hospital, 28 Fuxing Road, Beijing 100853, PR China

## Abstract

**Background:**

To explore the pathological changes of three-dimension structure of ventricular myocardial fibers after anterior myocardial infarction in dog heart.

**Methods:**

Fourteen acute anterior myocardial infarction models were made from healthy dogs (mean weight 17.6 ± 2.5 kg). Six out of 14 dogs with old myocardial infarction were sacrificed, and their hearts were harvested after they survived the acute anterior myocardial infarction for 3 months. Each heart was dissected into ventricular myocardial band (VMB), morphological characters in infarction region were observed, and infarct size percents in descending segment and ascending segment were calculated.

**Results:**

Six dog hearts were successfully dissected into VMB. Uncorresponding damages in myocardial fibers of descending segment and ascending segment were found in apical circle in anterior wall infarction. Infarct size percent in the ascending segment was significantly larger than that in the descending segment (23.36 ± 3.15 (SD) vs 30.69 ± 2.40%, P = 0.0033); the long axis of infarction area was perpendicular to the orientation of myocardial fibers in ascending segment; however, the long axis of the infarction area was parallel with the orientation of myocardial fibers in descending segment.

**Conclusions:**

We found that damages were different in both morphology and size in ascending segment and descending segment in heart with myocardial infarction. This may provide an important insight for us to understand the mechanism of heart failure following coronary artery diseases.

## Background

Postinfarct ventricular remodeling (PIVR) is the major cause of heart failure following coronary artery disease [1]. Microcosmically, PIVR has been recognized on molecular and genetic levels. Macroscopically, studies on PIVR has been limited to ventricular wall attenuation, chambers dilation and ventricular wall hypertrophy in unification area and so on [2,3]. However, few studies have been done on three-dimension structure of ventricular myocardial fibers after myocardial infarction. Torrent's hypothesis [4-17], ventricular myocardial band (VMB) theory, more reasonably elucidates three-dimension structure of myocardial fibers and the interaction of form with function in heart. According to VMB theory, cardiac ejection and filling function will be compromised whatever causes myocardial fiber damages in ascending or descending segment of heart [8]. In our previous study, we explored three-dimension architecture of myocardial fibers and sequential contractile function of ventricular myocardial band in the healthy hearts of pigs and humans [9-12]. In present study, we have further studied three-dimensional structural changes in ventricular myocardial fibers after myocardial infarction.

## Methods

### Experimental preparation: Establishing the model of acute myocardial infarction in dog [4]

Fourteen dogs received humane care in compliance with the 1996 NRC Guide for the Care and Use of Laboratory Animals. They were offered by Animal Experimental Center of PLA General Hospital. 14 dogs (16.5 to 19.0 kg) were premeditated with Ketamine hydrochloride (15 mg/kg) and diazepam (0.5 mg/kg) intramuscularly and were anesthetized with Pentobarbital sodium (10 mg/kg) and Norcuron (0.03 mg/kg). Support with a volume-controlled ventilator (Servo 900C, Siemens-Elema, Sweden) was maintained after tracheal intubation. The left femoral artery was cannulated for arterial pressure measurement. The electrocardiogram was monitored. Each dog underwent thoracotomy through the fifth intercostal space and the heart was exposed with pericardial incision. The left anterior descending branch was ligated by a 4-0 Prolene thread at the site between the first and second diagonal branch. Asynersis was found in the left ventricular anterior wall which appeared dark and the electrocardiogram showed classic acute myocardial infarction. Chest was closed after the vital signs were observed for an hour. Dietary activities were observed every day after surgery. Postoperative echocardiography was performed, and cardiac morphology and function were measured at 3 months.

### Experimental protocol

#### Anatomy of ventricular myocardial band

Six surviving dogs were sacrificed and their hearts were harvested at 3 months. Each heart was treated and dissected by hand with the method described by Torrent-Guasp [5].

#### Evaluation of infarction

Double helical VMB was unfolded. Morphologic characteristics were determined. In addition, to measure the infarct size more exactly, we calculated infarct size percents of descending segment and ascending segment respectively with a new method which was designed based on Torrent's double helical VMB theory rather than a traditional method.

As the VMB was unfolding naturally, it was photographed with a digital camera and the pictures were fed into the computer. The infarct sizes in descending segment and ascending segment were measured using picture processing software (Sichuang Company). Then, the infarct size percents of descending segment and ascending segment were calculated respectively as follows:

ISPDS=100%×IADS/ADS

ISPAS=100%×IAAS/AAS

ISPDS: Infarct size percent of descending segment; IADS: Infarct area in descending segment; ADS: area of descending segment; ISPAS: Infarct size percent of ascending segment; IAAS: Infarct area in ascending segment; AAS: area of ascending segment.

### Statistical analysis

SPSS 10.0 was used for statistical analysis. (Statistical analysis was performed using SPSS 10.0.) Infarct size data were compared by t-test between two segments and were reported as mean ± standard deviation (mean ± SD). P-values < 0.05 were considered statistically significant.

## Results

### Echocardiography

Echocardiography confirmed that old myocardial infarction was successfully established in the 6 dogs, in which dyskinesia was found in the left anterior wall and apex. Postinfarct left ventricular end-diastolic dimension (LVEDD) was larger than that of normal heart (34.3 ± 7.8 (SD) vs 25.6 ± 7.3 mm, P = 0.106). Postinfarct ejection fraction (EF) was significantly smaller than that of normal heart (45.7 ± 4.5 (SD) vs 59.8 ± 5.2%, P = 0.0018.)

### Morphologic characteristics of VMB

**Figure 1:**
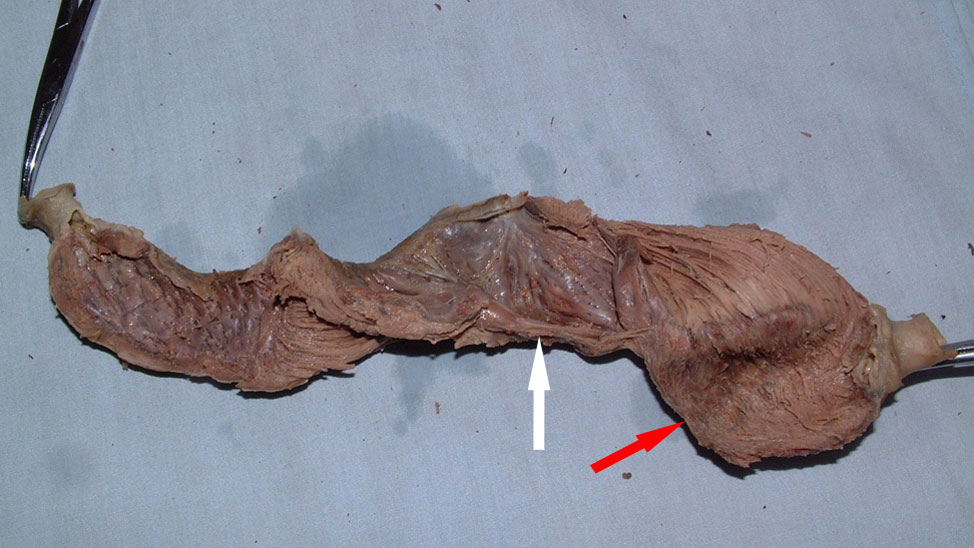
**Totally unfolded VMB with old myocardial infarction: white arrow indicated descending segment and red arrow indicated ascending segment**.

**Figure 2:**
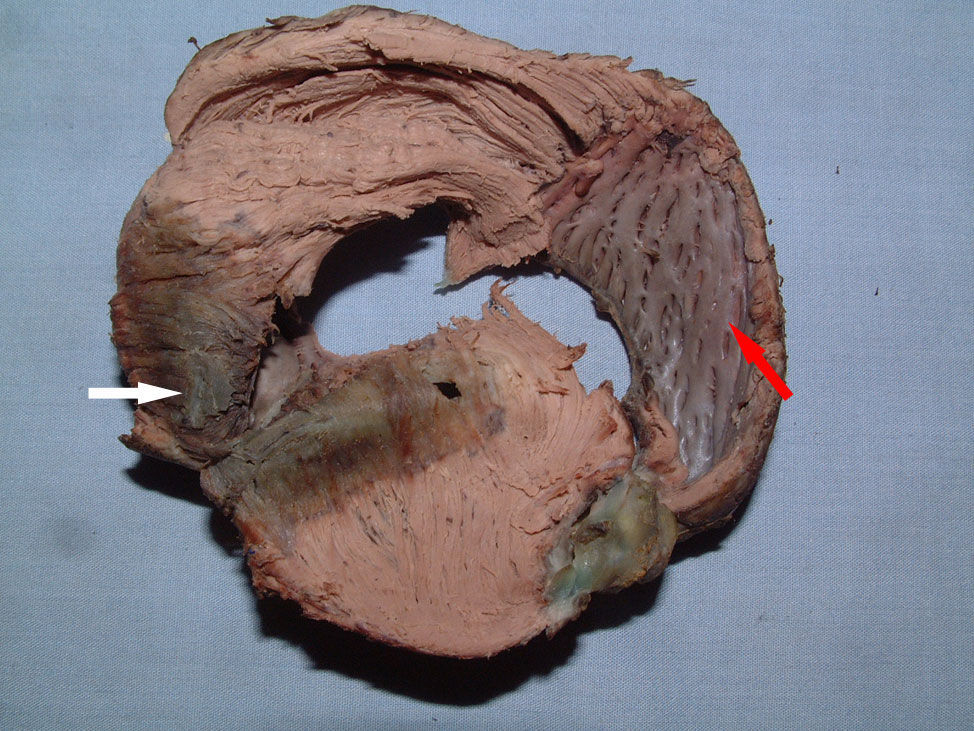
**Partially unfolded VMB mainly showed infarction region in apical circle**. White arrow indicated infarction region in descending segment and red arrow indicated ascending segment. (Anterior wall infarction led to uncorresponding damages in ascending and descending segment).

Six hearts were successfully dissected into ventricular myocardial band (VMB) (Figure 1), which was composed of basal and apical loops as Torrent-Guasp described [5]. Basal loop of the unraveled band contained transverse fibers that wrapped around the right and left ventricles (Figure 2). Apical loop contained oblique fibers that were comprised of descending and ascending segments (Figure 2).

**Figure 3:**
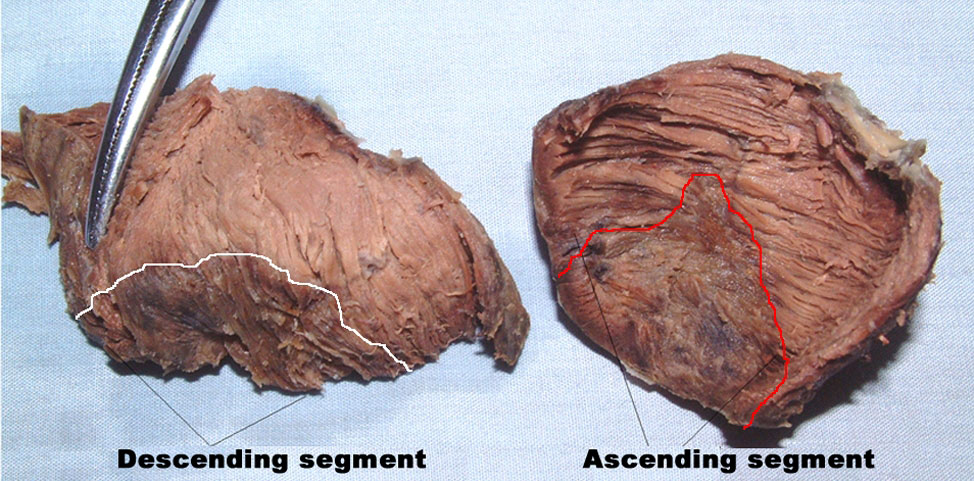
**Apical circle was divided into descending segment and ascending segment**. White-line-marked area indicated infarction region in descending segment and red-line-marked area indicated infarction region in ascending segment. The damage in the ascending was greater than that in the descending segment.

**Figure 4:**
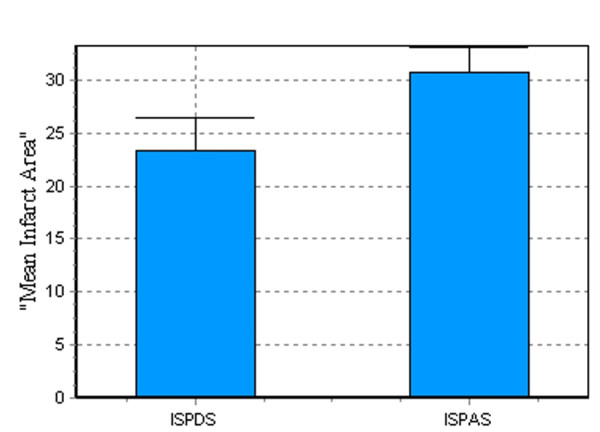
**Comparion of ISPDS and ISPAS**.

Anterior wall infarction mainly involved apical loop, but the damages in ascending and descending segments appeared uncorresponding (Figure 3). Infarction size percent of ascending segment (ISPAS) was significantly larger than that of descending segment (ISPDS) (23.36 ± 3.15 (SD) vs. 30.69 ± 2.40%, P = 0.0033, Figure 4) and long axis of infarction region was perpendicular to the orientation of myocardial fibers in ascending segment. However, long axis of infarction region was parallel with the orientation of myocardial fibers in descending segment. (Figures 2 and 3)

## Disscussion

Postinfarct ventricular remodeling (PIVR) is the major pathologic basis of chronic heart failure following myocardial infarction. It always occurs regardless of the degree of infarction and involves myocardial fibers in both infarct and non-infarct regions. Its main macropathologic changes are ventricular wall attenuation, chamber dilation and ventricular wall hypertrophy in non-infarct region and so on. In addition, these changes can lead to chronic heart failure and ventricular aneurysm. Torrent [4-7] described VMB as elementary cardiac structure that is composed of double helical coil named basal and apical loops. The basal loop contains right and left segments. The apical loop, which includes descending and ascending segments, is the (The apical loop includes descending and ascending segments. This is) material basis of cardiac pumping function with high efficiency. Whatever causes damage in VMB will inevitably impair ejection and filling function [8].

Measurement of infarct size percent is one of the important methods for evaluating PIVR. In present study, we found that anterior wall infarction led to uncorresponding damages in the apical loop. Infarct size percent of ascending segment was significantly larger than that of descending segment and we found that long axis of infarction region was perpendicular to the orientation of myocardial fibers in ascending segment, where the major myocardial fibers were broken and long axis of infarction region was parallel with the orientation of myocardial fibers in descending segment, where only partial myocardial fibers disappeared. We found that damages were different in both morphology and size in ascending segment and descending segment in heart with myocardial infarction.

It has been recently reported that postinfarcted filling function decrease was an independent risk factor of congestive heart failure and death in patients with myocardial infarction [13,14]. However, the mechanism is unclear so far [15]. Some researchers claimed that postinfarct scarring and diffuse myocardial fibrosis probably caused the damage of diastolic function [16,17]. Certainly, this can interpret why cardiac diastolic function decrease in long-term period after myocardial infarction. Therefore, previous studies can't interpret why diastolic function decrease shortly after myocardial infarction. In our study, we found that there were damages in ascending and descending segments in heart with anterior wall infarction. According to Torrent's hypothesis, descending segment contraction is the main force for ventricle ejection and ascending segment contraction is the main force for ventricle filling during 'isovolumetric relaxation' phase of diastole [8]. Our results indicated greater damages in ascending segment than those in descending segment. These pathologic changes may justify the mechanism of diastolic function disorder in heart with myocardial infarction. Different studies on the relationship between postinfarcted diastolic function and prognosis have reached a uniform conclusion that long-term death risk will increase if postinfarcted left ventricular filling pressure increases [13,14,18]. Generally, postinfarcted diastolic function disorder is often associated with systolic function disorder in clinical cases. Many patients had mild systolic function disorder, but obvious diastolic function disorder [2]. In present study, we found that anterior wall infarction involved less damage in descending segment. Castella and colleagues have suggested that asynchronous shortening of the endocardium and epicardium characterized by prolonged contraction of the descending segment may be a principal factor of diastolic dysfunction. This may explain the mild systolic function disorder in clinical patient, because descending segment is responsible for the main force for ventricle ejection.

In present study, we conclude that damages were different in both morphology and size in ascending segment and descending segment in heart with myocardial infarction. This may provide an important insight for us to understand the mechanism of heart failure following coronary artery diseases.

## Competing interests

The authors declare that they have no competing interests.

## Authors' contributions

**Gao C: **Study design, development of methodology, collection and analysis of data, writing the manuscript and supervision. **WHY: **Completion of the experiment. **LBL: **Completion of the experiment. All the authors have read and approved the final manuscript.

## Authors' information

Professor Changqing Gao is Chairman and professor of the Department of Cardiovascular Surgery, Director of the Minimally Invasive and Robotic Cardiac Surgery Center, PLA General Hospital, Beijing, China, and Director of the Institute of Cardiac Surgery, and chief surgeon. His professional interests include acquired heart disease, mitral and aortic valve repair/replacement, aneurysms of the thoracic aorta, and heart transplantation. He has a special interest in complex coronary artery bypass, off-pump coronary artery bypass, left ventricular aneurysms, and minimally invasive cardiac surgery
